# Hémogramme chez les hypertendus vus au laboratoire du CHU-HJRB d'Antananarivo en 2013

**DOI:** 10.11604/pamj.2016.23.49.8900

**Published:** 2016-02-19

**Authors:** Zafindrasoa Domoina Rakotovao-Ravahatra, Fidiniaina Mamy Randriatsarafara, Fetralinjiva Razafimanantsoa, Felana Ranaivo Rabetokotany, Andriamiadana Luc Rakotovao

**Affiliations:** 1 Unité Laboratoire du CHU-HJRB d'Antananarivo, Madagascar; 2 Département Santé publique Faculté de Médecine d'Antananarivo, Madagascar; 3 Service de Laboratoire du CHU de Toamasina, Madagascar

**Keywords:** Hémogramme, HTA, anémie, hyperleucocytose, diabète, Blood count, high blood pressure, anaemia, leucocytosis, diabetes

## Abstract

**Introduction:**

L'hémogramme est un bilan biologique de routine demandé chez tout patient souffrant d'Hypertension Artérielle (HTA). Cette étude se propose de décrire les résultats d'hémogramme chez les hypertendus et d'identifier les pathologies associées.

**Méthodes:**

Il s'agit d'une étude rétrospective type descriptif s’étalant du 01 Décembre 2012 au 31 Décembre 2013 au laboratoire du Centre Hospitalo-Universitaire Hôpital Joseph Raseta Befelatanana (CHU-HJRB) d'Antananarivo. Tous les registres des résultats des hypertendus demandant un hémogramme ont été exploités.

**Résultats:**

Parmi les 151 hypertendus, 91 (60,3%) ont présenté des hémogrammes pathologiques. Parmi ces derniers, 64 (70,4%) ont montré un seul type d'anomalie et 27 (29,6%) des anomalies multiples. Les anémies (33,91%), les hyperleucocytoses (33,04%), les polyglobulies (10,43%) et les leucopénies (9,57%) sont les plus fréquentes. Pour les anomalies multiples, les anémies associées aux hyperleucocytoses sont les plus observées (29,6%). Les anémies microcytaires (41%) et les hyperleucocytoses à polynucléaires neutrophiles sont les plus dominantes (47,4%). Les patients hospitalisés en néphrologie (90%) et en endocrinologie (81,3%) sont les plus concernés (p = 0,008). Les hypertendus moins de 20 ans (100%) et les femmes (61,5%) sont les plus affectés (p > 0,05). Les crises convulsives (100%), les œdèmes des membres inférieurs (100%) et le diabète (70%) sont les signes et pathologies associés les plus rencontrés (p > 0,05).

**Conclusion:**

L'hémogramme doit être prescrit chez tout patient hypertendu pour connaître les affections sous-jacentes qui seront traitées simultanément avec l'HTA. Ainsi, le patient hypertendu sera pris en charge convenablement et son espérance de vie sera améliorée.

## Abstract (en)

**Introduction:**

The blood count is a routine laboratory test prescribed to patients suffering from high blood pressure. This study aims to describe the results of blood count from hypertensive patients and to identify conditions associated.

**Methods:**

This retrospective and descriptive study concerns the period from the 1st of December 2012 to 31th of December 2013 in the laboratory of University Hospital Center CHU-HJRB of Antananarivo. All records of results from hypertensive patients who requested blood count have been exploited.

**Results:**

Among 151 hypertensive patients, 91 (60.3%) had pathological blood counts. Of these, 64 (70.4%) showed one type of abnormality and 27 (29.6%) had multiple abnormalities. The anaemia (33.91%), the leukocytosis (33.04%), the polycythemia (10.43%) and the leukopenia (9.57%) are the most frequent. Among the multiple abnormalities, anaemia associated with leukocytosis is the most observed (29.6%). The microcytosis anaemia (41%) and the neutrophil polymorphonuclear leukocytosis are the most dominant (47.4%). Patients in nephrology (90%) and in endocrinology (81.3%) are the most concerned (p = 0,008). Hypertensive under 20 years old (100%) and women (61.5%) are the most affected (p > 0, 05). The seizures (100%), the edema of the lower limbs (100%) and diabetes (70%) are the signs and pathology most encountered.

**Conclusion:**

The blood count should be prescribed for all hypertensive patients to identify the affections that will be treated simultaneously with the high blood pressure. Thus, hypertensive patients will have a good follow-up and their life expectancy will be improved.

## Introduction

L'Hypertension artérielle (HTA) est un problème de santé publique mondiale. Selon l'Organisation Mondiale de la Santé (OMS), les maladies cardio-vasculaires sont responsables d'environ 17 millions de décès par an dans le monde, soit près d'un tiers de la mortalité totale. Sur ce chiffre, 9,4 millions de morts par an sont imputables aux complications de l'hypertension [1]. A Madagascar, la prévalence de l'HTA chez les adultes de25 ans et plus est de 40,6% chez l'homme et 37,5% chez la femme selon la statistique sanitaire de l'OMS en 2014 [2]. Selon les données recueillies auprès du Service de Statistique du Ministère de la Santé Publique Malgache en 2013, au niveau des Centres Hospitaliers de Référence, l'HTA essentielle représente la deuxième cause des pathologies médicales les plus fréquemment rencontrées avec 6,4% des consultations externes. La létalité liée à l'HTA vue au niveau des Centres Hospitaliers de Référence s’élève à près de 5,5% [3]. Les facteurs de risque de l'HTA sont nombreux, principalement l’âge, le sexe, le stress, les émotions, l'obésité, les habitudes alimentaires et toxiques, le manque d'activité physique et les maladies sous-jacentes comme le diabète [4, 5]. Des études sur les facteurs de risque, les causes et les complications de l'HTA devraient être faites régulièrement pour améliorer la prévention de l'HTA et la prise en charge des patients hypertendus. L'hémogramme est un bilan biologique de routine qui permet de connaître les valeurs érythrocytaires, leucocytaires et plaquettaires de l'individu ainsi que les caractéristiques morphologiques de ces cellules. Les données obtenues par cet examen sont interprétées en fonction des repères ou valeurs de référence qui dépendent de l’âge, du genre ou de l’état physiologique de l'individu [6]. De même, en cas de déséquilibre ou de maladie sous-jacente chez un individu, on peut observer beaucoup de perturbations dans les résultats d'hémogramme. D'où l'objectif général de cette étude qui consistait à décrire les résultats d'hémogramme chez les hypertendus. Les objectifs spécifiques étaient de: (i) identifier les différentes anomalies et les pathologies associés à l'HTA selon les résultats d'hémogramme (ii) décrire les facteurs associés aux résultats d'hémogrammes chez l'hypertendu (iii) Proposer des suggestions concernant la prise en charge et le suivi des patients hypertendus.

## Méthodes

Il s'agit d'une étude rétrospective de type descriptif sur une période allant de 01 Décembre 2012 au 31 Décembre 2013 au laboratoire du CHU-HJRB d'Antananarivo. Tous les registres des résultats des patients hypertendus ayant demandé un hémogramme ont été exploités. Au sein de ce laboratoire, l'hémogramme a été effectué sur l'automate d'hématologie ABX Pentra 60 et le résultat a été enregistré dans le cahier de registre. Ceux qui contiennent des renseignements incomplets ont été exclus de l’étude. Les paramètres étudiés ont été l’âge, le genre, les renseignements cliniques, les services prescripteurs et les résultats d'hémogramme. L'hémogramme pathologique constitue le critère de positivité de la présente étude. Un hémogramme est dit pathologique lorsqu'il présente une ou plusieurs anomalies soit une augmentation soit une diminution des valeurs des éléments figurés du sang par rapport aux valeurs de référence des constantes biologiques. Etant donné qu'il n'y a pas encore de norme pour les valeurs normales de l'hémogramme de la population malgache, nous avons utilisé les valeurs de références proposées par l’équipe d'experts du groupe francophone d'hématologie cellulaire (GFHC) applicables à tous les laboratoires de biologie médicale en France Métropolitaine [7]. La saisie et le traitement des données ont été effectués sur le logiciel Epi-info 3.5.2. La comparaison des pourcentages a fait appel aux tests de Chi carré. Pour des impératifs d'ordre éthique, l'autorisation des responsables de l’établissement a été obtenue avant la collecte des données dans les registres. La saisie a été réalisée de façon anonyme pour garder la confidentialité. Le seuil de signification statistique à utiliser est de p= 0,05.

## Résultats

**Figure 1:**
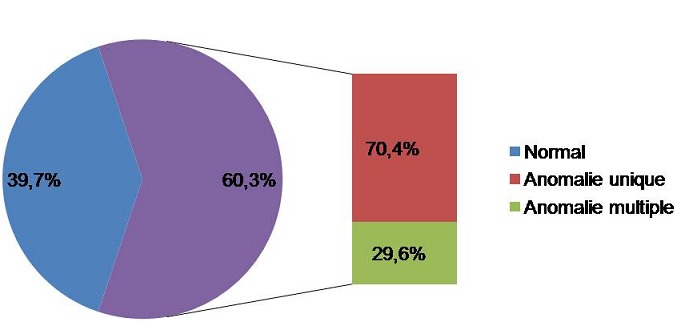
Répartition des hypertendus selon les résultats d'hémogramme

Au total, pendant la période d’étude, 151 patients hypertendus ont demandé un hémogramme. 91 patients ont présenté des hémogrammes pathologiques soit 60,3% des cas. Parmi ces derniers, 64 cas n'ont montré qu'un seul type d'anomalie (70,4%) et 27 cas ont présenté des anomalies multiples (29,6%) (Figure 1).

### Les différents résultats d'hémogrammes pathologiques

**Figure 2:**
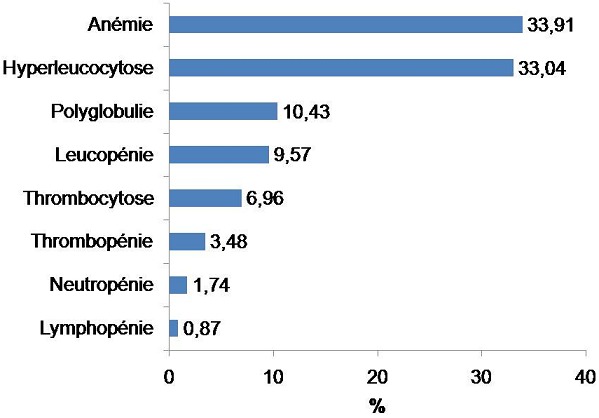
Résultats des hémogrammes pathologiques

Les anémies (33,91%) et les hyperleucocytoses (33,04%) sont les 2 grandes anomalies les plus rencontrées dans les hémogrammes pathologiques que les anomalies soient uniques ou multiples suivies des polyglobulies (10,43%) et des leucopénies (9,57%) (Figure 2). Parmi les 27 cas d'anomalies multiples, les anémies associées aux hyperleucocytoses sont les plus nombreuses (29,6%) (Tableau 1). Pour les anémies, celles qui sont microcytaires sont les plus dominantes (41%) suivies des anémies normocytaires (35,9%) et macrocytaires (23,1%). Pour les hyperleucocytoses, celles qui sont à polynucléaires neutrophiles sont les plus nombreuses (47,4%) suivies des éosinophilies (18,4%), des lymphocytoses (7,9%). Il a été noté que les hyperleucocytoses associant les 3 lignées en même temps représentent 26,3% des cas.

**Tableau 1:** Résultats des hémogrammes pathologiques selon les anomalies multiples

Anomalies multiples	Effectif	%
Anémie + hyperleucocytose	8	29,6
Anémie + thrombocytose	4	14,8
Anémie+ thrombopénie	3	11,1
Anémie + éosinophilie	2	7,4
Anémie + leucopenia	2	7,4
Anémie + lymphocytose	1	3,7
Anémie+ lymphopénie	1	3,7
Polyglobulie + thrombopénie	3	11,1
Hyperleucocytose + thrombocytose	1	3,7
Neutropénie + éosinophilie	1	3,7
Neutropénie + thrombopénie	1	3,7
Total	27	100

### Facteurs associés aux résultats d'hémogramme chez les hypertendus

Concernant les facteurs associés, les patients hospitalisés en néphrologie (90%) et en endocrinologie (81,3%) sont les plus concernés (p = 0,008). Les hypertendus moins de 20 ans (100%) et plus de 60ans (64,5%) ainsi que les femmes (61,5%) sont plus affectés par les hémogrammes pathologiques (p > 0,05). Pour les signes cliniques associées à l'HTA, les crises convulsives (100%), les œdèmes des membres inférieurs (100%) et le diabète (70%) sont les plus rencontrés (p > 0,05, NS) (Tableau 2).

**Tableau 2:** Facteurs associés aux résultats d'hémogramme chez les hypertendus

Facteurs associés	Hémogramme pathologique		Hémogramme normal		
	n = 91		n = 60		p
	Effectif	%	Effectif	%	
**Tranches d’âge**					
0-19 ans	3	100	0	0	0,37
20-39 ans	8	57,1	6	42,9	
40-59 ans	40	55,6	32	44,4	
60 ans et plus	40	64,5	22	35,5	
**Genre**					
Masculin	32	58,2	23	41,8	0,69
Féminin	59	61,5	37	38,5	
**Services**					
Cardiologie	33	63,5	19	36,5	0,008
Rhumato-dermato	16	51,6	15	48,4	
Endocrinologie	13	81,3	3	18,7	
Neuro-psychiatrie	11	73,3	4	26,7	
Nephrologie	9	90	1	10	
Externe	6	37,5	10	62,5	
Autres	3	27,3	8	72,7	
**Signes cliniques et pathologies associés**					
HTA seule	75	59,1	52	40,9	0,64
Diabète	7	70	3	30	
AVC	2	66,7	1	33,3	
Dyspnée	1	33,3	2	66,7	
OMI	2	100	0	0	
Crises convulsive	2	100	0	0	
Autres	2	50	2	50	

## Discussion

Dans la présente étude, 60,3% des hypertendus ont présenté une ou plusieurs anomalies dans leur résultat d'hémogramme. Cette proportion est non négligeable car elle témoigne déjà des effets néfastes de l'HTA sur l'organisme humain. De même, ces anomalies peuvent être révélatrice d'autres affections sous-jacentes qu'il est possible de diagnostiquer par d'autres investigations complémentaires. Concernant les anomalies rencontrées, l'anémie est la plus fréquente. En effet, l'anémie est une affection fréquente en milieu hospitalier. Elle est liée au régime alimentaire, au mode de vie, à l’état physiologique de l'individu; elle est souvent associée à l'HTA [8, 9]. D'une part, la prédominance de cette anémie chez les hypertendus doit être prise en compte car elle peut nécessiter déjà une transfusion même si le taux d'hémoglobine est encore à 8 g/dl chez les sujets âgés avec des antécédents cardio-vasculaires [10]. D'autre part, l'anémie peut être révélatrice d'une insuffisance rénale qui est une complication grave de l'HTA. Dans ce cas, l'anémie est due à un déficit de production d'erythropoiétine endogène (EPO) par les reins [11]. En effet, l'EPO est une hormone qui stimule la production des globules rouges dans la moelle osseuse [12]. Ainsi, son déficit ou son absence entraine une anémie à long terme justifiant le suivi régulier de l'hémogramme des patients hypertendus.

L'hyperleucocytose vient au deuxième rang après l'anémie. En effet, ce n'est pas l'HTA qui entraine cette anomalie mais d'autres affections ou maladies sous-jacentes associées. Les leucocytes ont tendance à augmenter lorsque le sujet présente une infection ou une réaction inflammatoire [13]. Dans ce cas, il s'agit d'une hyperleucocytose réactionnelle. Un simple traitement de ces éventuelles infections entraine rapidement la normalisation du nombre des globules blancs. Néanmoins, le pronostic est plus sévère chez les patients ayant une leucocytose très élevée [14]. En effet, cette dernière peut être révélatrice d'une maladie grave comme les hémopathies malignes [15]. Aussi est-il nécessaire de faire d'autres investigations complémentaires devant une hyperleucocytose très élevée chez un hypertendu pour diagnostiquer ces éventuelles pathologies associées. La polyglobulie représente la 3^ème^anomalie rencontrée. D'autres études ont également montré la fréquence de cette anomalie chez les hypertendus [16–18]. D'une part, cette polyglobulie entraine une hypertension artérielle en augmentant la viscosité du sang et en ralentissant le flux sanguin. D'autre part, ce ralentissement va favoriser la formation des caillots sanguins aboutissant aux thromboses artério-veineuses. A leur tour, ces thromboses seront à l'origine des nombreuses complications cardio-vasculaires de l'HTA tels que l'Infarctus du Myocarde et l'Accident Vasculaire Cérébral (AVC) [19]. Et Inversement, l'HTA constitue également un facteur de risque de polyglobulie [20].

Les associations anémie-hyperleucocytose et anémie-thrombocytose sont les anomalies multiples les plus fréquentes. D'habitude, les cliniciens se penchent plutôt vers les bilans lipidiques et rénaux étant donné que l'HTA fait partie des maladies de surcharge. Pourtant, le suivi systématique de l'hémogramme permet de prévenir des situations graves telles les thromboses artério-veineuses secondaires, entre autres, aux thrombocytoses [21]. Concernant les types d'anémie, celles qui sont microcytaires sont les plus fréquentes dans la présente étude. Les anémies microcytaires, généralement due à une carence en fer est la plus fréquente dans tous les pays quels que soit les pathologies associées [22]. Ce sont les sujets âgés anémiés et hypertendus qui sont les plus vulnérables avec un risque d'apparition des complications cardio-vasculaires. D'ailleurs, une étude a montré que le pronostic vital et/ou fonctionnel des sujets âgés souffrant d'insuffisance cardiaque et de maladie coronarienne est directement et significativement lié aux valeurs d'hémoglobine [23]. Concernant les types d'hyperleucocytose, celles qui sont à Polynucléaires neutrophiles sont les plus nombreuses. L'hyperleucocytose à PNN est un signe banal de toute infection ou de tout syndrome inflammatoire aigu ou chronique. Aussi est-elle fréquente dans les nécroses tissulaires aiguës comme le syndrome coronarien aigue ou l'Infarctus du myocarde qui sont des complications vasculaires de l'HTA [14]. Concernant les facteurs associés aux résultats des hémogrammes chez les hypertendus, les femmes hypertendues sont les plus affectées par les hémogrammes pathologiques. Cela démontre que les femmes sont plus vulnérables. De même, d'autres études soulignent que l'HTA apparaît plus rapidement chez le genre féminin [24, 25]. Les hémogrammes pathologiques sont fréquents dans les âges extrêmes de la vie notamment chez les hypertendus moins de 20 ans et plus de 60ans. Chez le sujet jeune, l'HTA peut être secondaire à une pathologie sous-jacente telle que l'hyperaldostéronisme primaire [26]. Les troubles de l'hémogramme relèvent des dites pathologies et non de l'HTA elle-même. D'autres examens de laboratoire vont permettre d'identifier ces étiologies. Chez le sujet âgé, ces hémogrammes pathologiques peuvent être secondaires au vieillissement de l'organisme majoré par les facteurs de risque cardio-vasculaires associés. De même, l'HTA est secondaire aux modifications de la structure artérielle et de sa fonction observées avec le vieillissement. Les grosses artères deviennent rigides et contribuent à l'augmentation de la vitesse de l'onde de pouls responsable d'une HTA systolique [27]. Les patients hospitalisés présentent significativement des hémogrammes pathologiques en particulier dans les services de néphrologie et d'endocrinologie. En effet, l'insuffisance rénale en service de néphrologie est une complication assez fréquente de l'HTA [28]. Cette insuffisance rénale est souvent associée à l'anémie qui est une anomalie fréquente de l'hémogramme [29, 30]. De même, le diabète qui est une maladie fréquente en service d'endocrinologie, est souvent associé à l'HTA avec un risque cardio-vasculaire élevé [31]. Il est également responsable d'Insuffisance rénale et d'anémie à long terme [32]. Concernant les signes cliniques et pathologies associés, on constate que les sujets hypertendus présentent souvent des hémogrammes pathologiques au stade de complications. Ces complications sont représentées fréquemment par les complications cardio-vasculaires (œdèmes des membres inférieurs, AVC), neurologiques (crises convulsives). Ces hémogrammes pathologiques s'observent également en cas d'association de l'HTA avec le diabète. D'où l'intérêt de l'hémogramme qui va permettre d’établir le pronostic, de guider ou de modifier le traitement anti - hypertenseur afin d'assurer une meilleure prise en charge du sujet hypertendu. En bref, nous suggérons aux cliniciens d'effectuer un bilan biologique complet comportant obligatoirement un hémogramme chez tout patient hypertendu pour connaître les éventuelles affections sous-jacentes qui seront traitées simultanément avec l'HTA. Ces examens doivent être effectués aussi bien pour le bilan biologique initial que pour le bilan de suivi systématique. Ainsi, le patient hypertendu sera pris en charge convenablement et son espérance de vie sera améliorée.

## Conclusion

De nombreuses anomalies de l'hémogramme ont été détectées chez la majorité des patients hypertendus. Aussi, l'hémogramme chez l'hypertendu est-il un bilan biologique essentiel à prescrire obligatoirement au même titre que les bilans lipidiques et les autres bilans de surcharge. Les variations qualitatives et quantitatives de ces anomalies d'hémogramme révèlent la gravité et le pronostic de l'HTA ainsi que les éventuelles pathologies sous-jacentes. La connaissance de ces anomalies permet de renforcer, d'ajuster ou de modifier les traitements médicamenteux de l'hypertendu. De même, le traitement des maladies sous-jacentes va améliorer le pronostic à cours et à losg terme de l'HTA permettant ainsi une bonne prise en charge des patients.

### Etat des connaissance sur le sujet

- En cas d'Hypertension Artérielle, les cliniciens se penchent surtout vers les bilans de surcharge car l'HTA fait partie des maladies de surcharge. De ce fait, l'hémogramme est souvent relégué en seconde position voire non prescrit par les cliniciens.
- L'insuffisance rénale responsable d'anémie (anomalie fréquente de l'hémogramme) est une complication fréquente de l'Hypertension Artérielle d'où l'intérêt de faire un hémogramme de routine chez les hypertendus pour surveiller les complications.

### Contribution de notre étude a la connaissance

- Mise en évidence de nombreuses anomalies de l'hémogramme chez les hypertendus qui révèlent des complications, des facteurs de risques et des pathologies associées à l'HTA.
- Intérêt de l'hémogramme chez les hypertendus qui devrait être prescrit par le clinicien au même titre que les bilans de surcharge.
- Amélioration de la prise en charge et du suivi des patients hypertendus.
